# Supplementary material for: SLC16A6 is a tyrosine transporter for the melanosome
Source: bioRxiv. 2026 Jul 7:2026.07.06.736842. Preprint. [Version 1] doi: 10.64898/2026.07.06.736842 (PMC13370447; doi:10.64898/2026.07.06.736842)
Supplement: Supplement 2 [file NIHPP2026.07.06.736842v1-supplement-2.pdf]

bioRxiv preprint doi: https://doi.org/10.64898/2026.07.06.736842; this version posted July 7, 2026. The copyright holder for this preprint (which was not certified by peer review) is the author/funder, who has granted bioRxiv a license to display the preprint in perpetuity. It is made available under a CC-BY 4.0 International license.

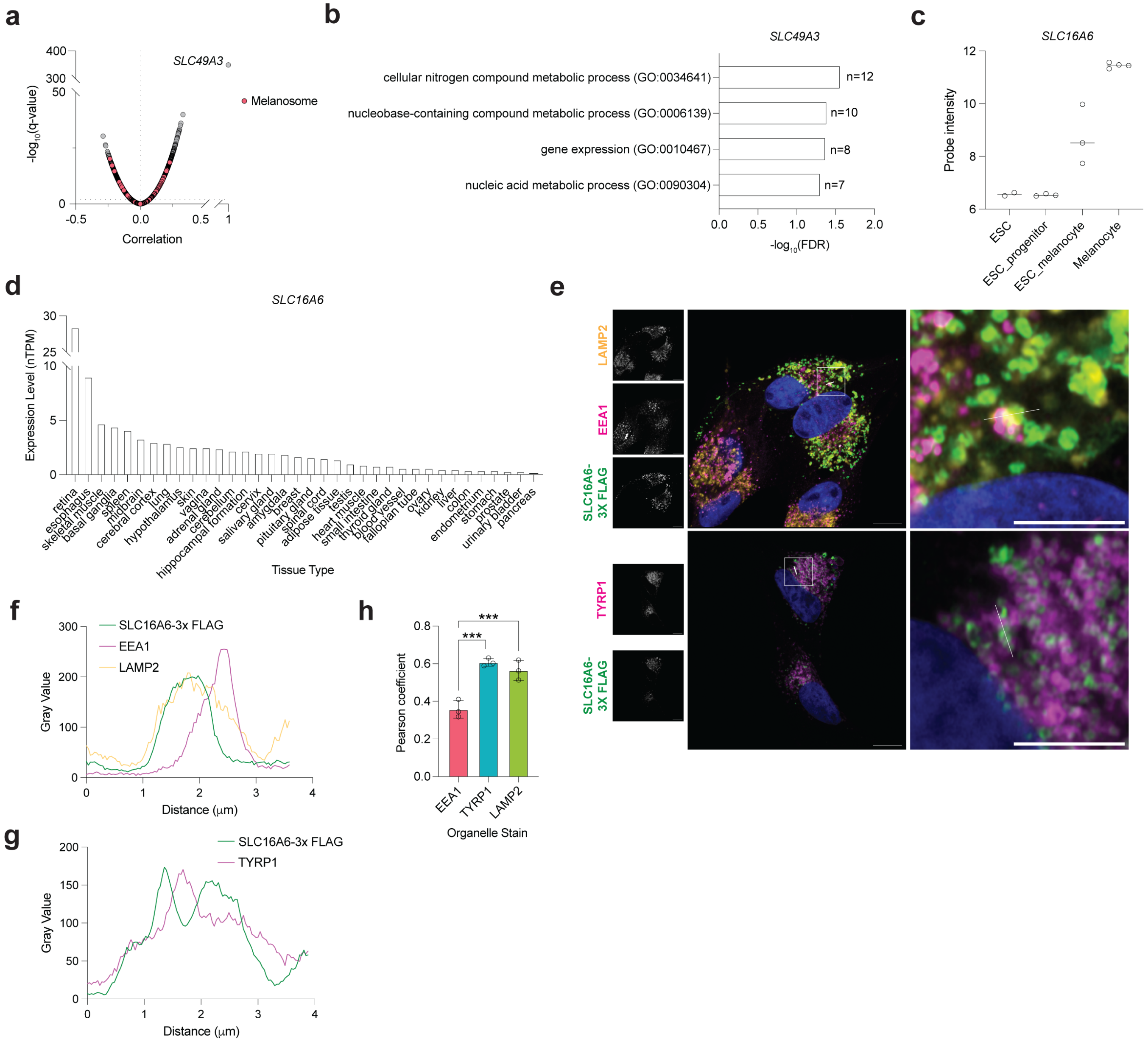

Figure S1. SLC16A6 is highly expressed in melanocytes and localizes to melanosomes. (a) *SLC49A3* transcriptome-wide correlation. Co-expression analysis of *SLC49A3* transcripts versus all other transcripts using the DepMap short-read expression dataset (26Q1). **(b)** Panther GO-slim biological process analysis of top 500 most positively correlating transcripts from (a). **(c)** *SLC16A6* expression across embryonic stem cells (ESCs), ESC-derived melanocyte progenitors, ESC-derived mature melanocytes, and primary melanocytes. Data extracted from GSE45226[70]. **(d)** *SLC16A6* expression, plotted as normalized transcript expression values, extracted from Genotype-tissue expression database. **(e)** Representative confocal microscopy images of ectopic SLC16A6-3X-FLAG colocalization (n=3 biological replicates; scale bar 10 µm). **(f)** Representative line scan analysis of SLC16A6-3X FLAG colocalization with EEA1 and LAMP2; line is indicated in (e), top image. **(g)** Same as (f) but colocalization with TYRP1; line is indicated in (e), bottom image. **(h)** Average whole cell Pearson correlation coefficients of ectopic SLC16A6-3X FLAG with indicated organelle stains (30 cells counted per trial, ***$P < 0.001$, n=3 biological replicates).

bioRxiv preprint doi: https://doi.org/10.64898/2026.07.06.736842; this version posted July 7, 2026. The copyright holder for this preprint (which was not certified by peer review) is the author/funder, who has granted bioRxiv a license to display the preprint in perpetuity. It is made available under a CC-BY 4.0 International license.

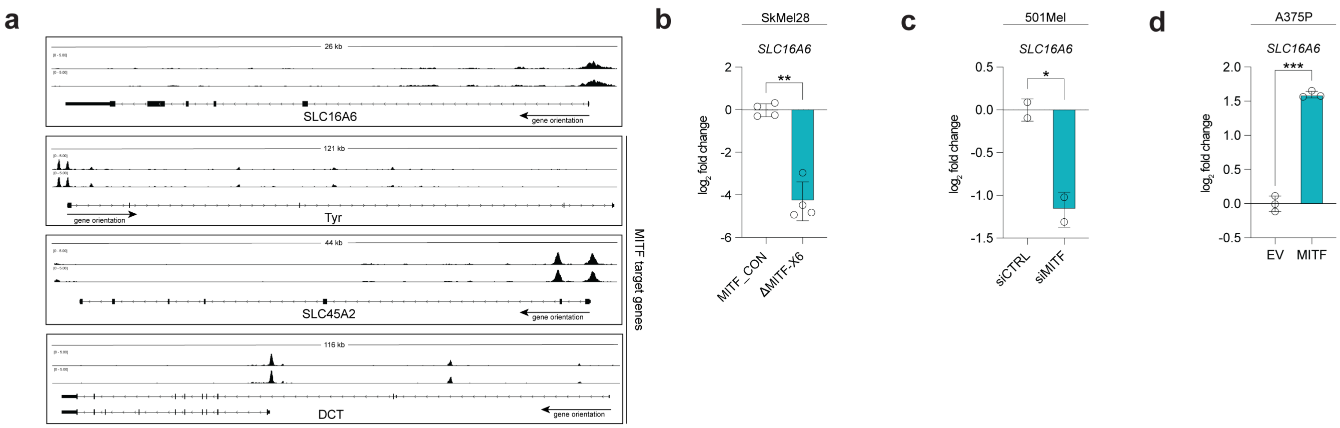

Figure S2. MITF binds the promoter region of *SLC16A6* to promote expression. (a) Genome browser tracks of indicated genes of MITF ChIP-Seq from GSE172383: SRX10640872 and SRX10640873. **(b)** RNA sequencing data of *SLC16A6* transcripts with a CRISPR generated *MITF* mutant (**$P < 0.01$)[27]. **(c)** As in (b), but with siRNA knockdown of *MITF* (*$P < 0.05$). **(d)** As in (b), but with overexpressed wildtype MITF (***$P < 0.001$).

bioRxiv preprint doi: https://doi.org/10.64898/2026.07.06.736842; this version posted July 7, 2026. The copyright holder for this preprint (which was not certified by peer review) is the author/funder, who has granted bioRxiv a license to display the preprint in perpetuity. It is made available under a CC-BY 4.0 International license.

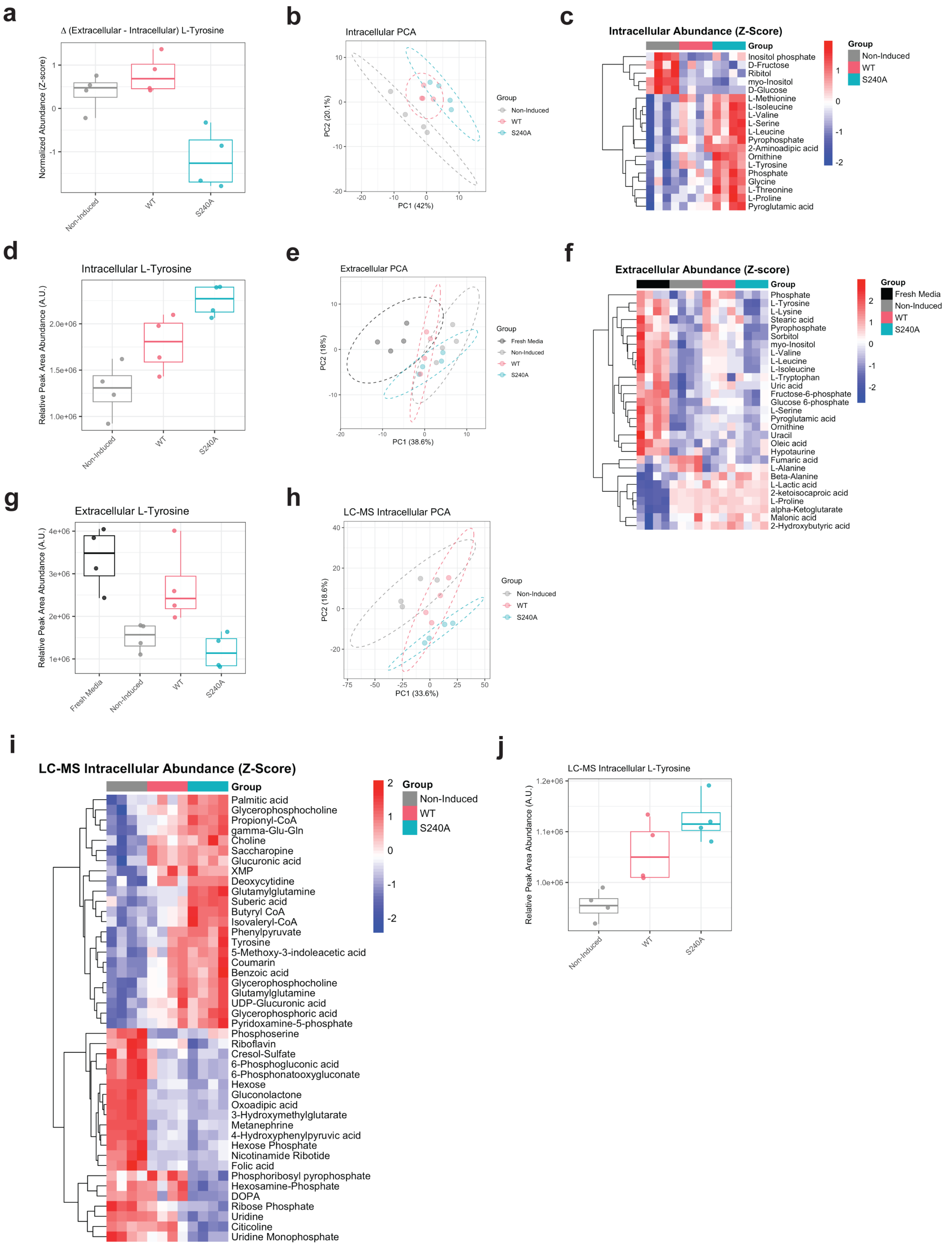

bioRxiv preprint doi: https://doi.org/10.64898/2026.07.06.736842; this version posted July 7, 2026. The copyright holder for this preprint (which was not certified by peer review) is the author/funder, who has granted bioRxiv a license to display the preprint in perpetuity. It is made available under a CC-BY 4.0 International license.

Figure S3. A plasma membrane-rerouted SLC16A6 alters tyrosine-derived metabolites abundances. (a) Boxplot of z-scored delta abundance for L-tyrosine measured by GC-MS. **(b-d)** Intracellular GC-MS dataset; **(e-g)** extracellular GC-MS dataset; **(h-j)** intracellular LC-MS dataset. PCA plots (b, e, h) show sample clustering. Heatmaps **(c, f, i)** show metabolites with significantly altered abundance by group. Metabolite abundances were log_2-transformed and z-score normalized prior to analysis. Boxplots **(d, g, j)** display relative L-tyrosine abundance measured as relative peak area (a.u.).

bioRxiv preprint doi: https://doi.org/10.64898/2026.07.06.736842; this version posted July 7, 2026. The copyright holder for this preprint (which was not certified by peer review) is the author/funder, who has granted bioRxiv a license to display the preprint in perpetuity. It is made available under a CC-BY 4.0 International license.

a

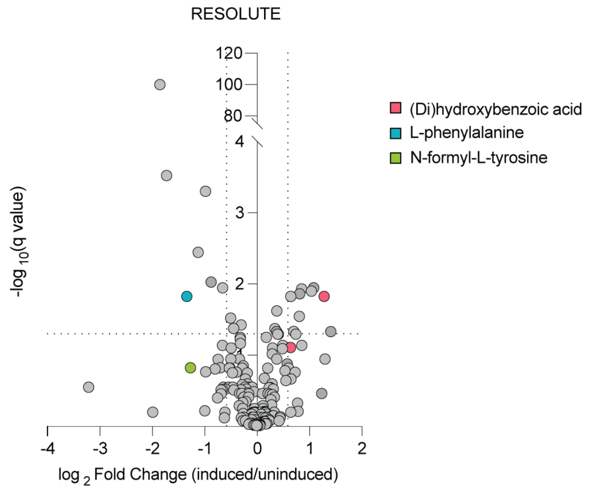

b

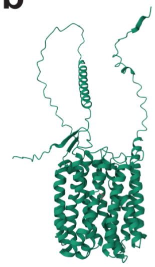

AlphaFill: O15403 (Monocarboxylate Transporter 7; SLC16A6)

**Compound**	**g-RMSd**
hydrocinnamic acid	1.82
lactate	1.79

Figure S4. SLC16A6 is predicted to transport aromatic metabolites. (a) RESOLUTE metabolomics data of HEK-293T cells induced WT SLC16A6 expression versus non-induced. Data extracted from re-solute.eu/knowledgebase/gene/SLC16A6. **(b)** AlphaFill substrate predictions for SLC16A6. *Left,* structure of SLC16A6 with hydrocinnamic acid. *Right*, table indicating two AlpahFill predicted substrates of SLC16A6.

bioRxiv preprint doi: https://doi.org/10.64898/2026.07.06.736842; this version posted July 7, 2026. The copyright holder for this preprint (which was not certified by peer review) is the author/funder, who has granted bioRxiv a license to display the preprint in perpetuity. It is made available under a CC-BY 4.0 International license.

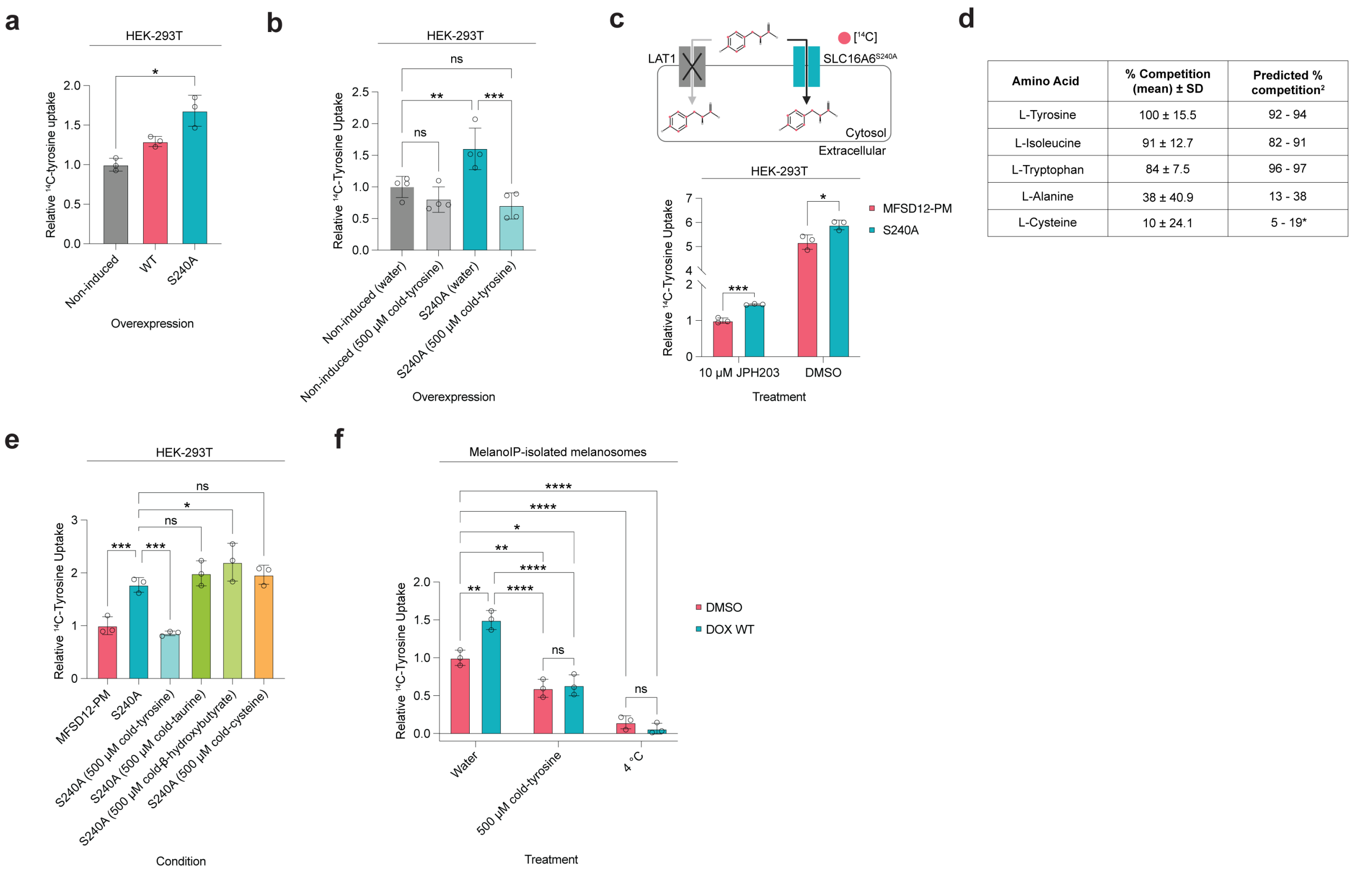

Amino Acid	% Competition (mean) ± SD	Predicted % competition[2]
L-Tyrosine	100 ± 15.5	92 - 94
L-Isoleucine	91 ± 12.7	82 - 91
L-Tryptophan	84 ± 7.5	96 - 97
L-Alanine	38 ± 40.9	13 - 38
L-Cysteine	10 ± 24.1	5 - 19*

Figure S5. SLC16A6 is sufficient to transport tyrosine. (a) Non-induced HEK-293T cells or HEK-293T cells expressing either WT SLC16A6 or SLC16A6^{S240A} were incubated in a buffer containing a LAT1 inhibitor. 5 µM cold-tyrosine and 1 µCi mL^{-1} ^{14}C-labeled tyrosine were added to initiate transport. Assay was performed for 10 minutes prior to washes and scintillation. Two-way ANOVA was used for statistical analysis. (*$P < 0.05$; n=3 biological replicates). **(b)** HEK-293T cells non-induced or induced SLC16A6^{S240A}-overexpression were incubated in a buffer containing LAT1 inhibitor, JPH203 at 10 µM. Cold-tyrosine (5 µM for transport assay; 500 µM for competition) and ^{14}C-labeled tyrosine were added to initiate transport. Assay was performed for 10 minutes prior to washes and scintillation. Two-way ANOVA was used for statistical analysis (**$P < 0.01$; n=4 biological replicates). **(c)** HEK-293T cells expressing either MFSD12-PM or SLC16A6^{S240A} were incubated in a buffer containing or lacking 10 µM JPH203. Cold-tyrosine (5 µM for transport assay; 500 µM for competition) and ^{14}C-labeled tyrosine were added to initiate transport. Assay was performed for 10 minutes prior to washes and scintillation. Two-way ANOVA was used for statistical analysis (*$P < 0.05$, ***$P < 0.001$, n=3 biological replicates). **(d)** Table comparison between mean percent competition in Fig. 3d versus reported percent competition[2]; asterisk denotes L-cystine competition metrics. **(e)** As in (Fig. 3d), but with cold-taurine, -β-hydroxybutyrate, -cysteine. Two-way ANOVA was used for statistical analysis (*$P <$ 0.05, ***$P < 0.001$; n=3 biological replicates). **(f)** As in (Fig. 3e), but with the addition of 500 µM cold-tyrosine or performed at 4°C for 10 min; samples normalized to 1% triton X-100 samples (*$P < 0.05$, **$P < 0.01$, ****$P < 0.0001$, n=3 biological replicates).

bioRxiv preprint doi: https://doi.org/10.64898/2026.07.06.736842; this version posted July 7, 2026. The copyright holder for this preprint (which was not certified by peer review) is the author/funder, who has granted bioRxiv a license to display the preprint in perpetuity. It is made available under a CC-BY 4.0 International license.

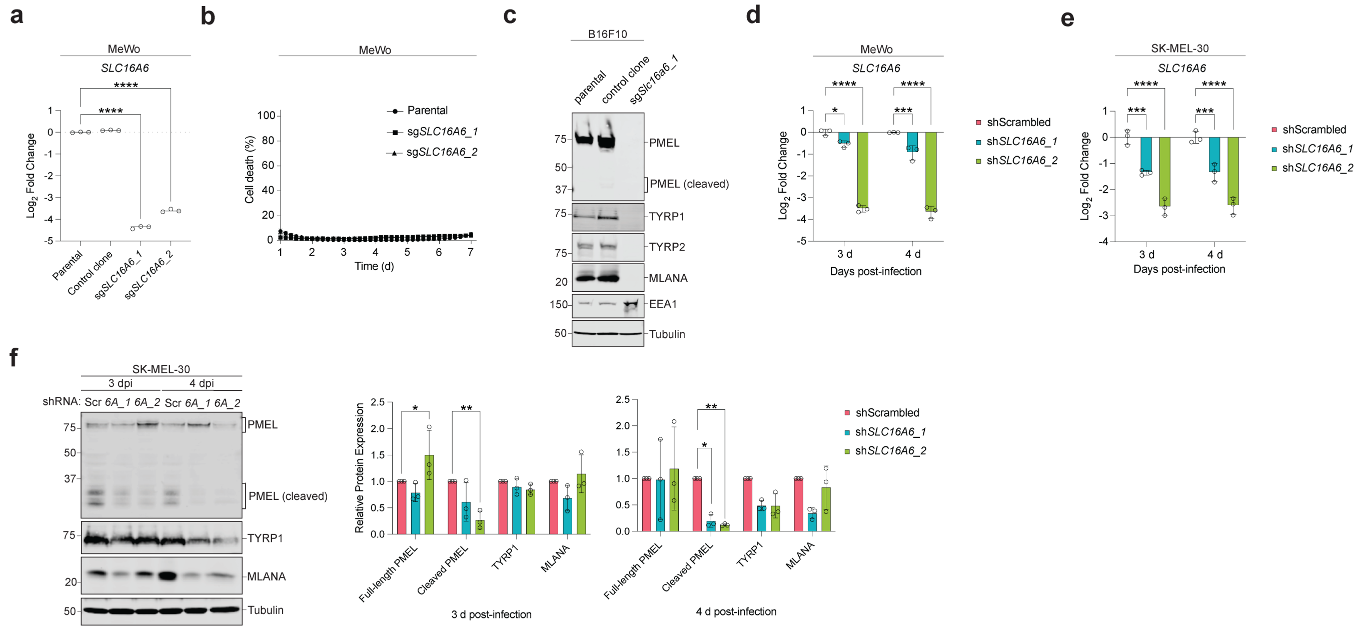

Figure S6. *SLC16A6* depletion downregulates melanosomal components. (a) *SLC16A6* transcript abundance changes in parental, control clone, and two *SLC16A6* knockout clones. **(b)** Cell death assay of parental and two *SLC16A6* knockout clones. **(c)** Representative immunoblots of indicated melanogenesis components in B16F10 parental, control clone, and one *SLC16A6* knockout clone (n=3 biological replicates). **(d)** qPCR of *SLC16A6* transcripts in MeWo melanoma cells three and four days post-infection with indicated shRNA lentiviruses. **(e)** qPCR of *SLC16A6* transcripts in SK-MEL-30 melanoma cells three and four days post-infection with indicated shRNA lentiviruses. **(f)** *Left,* representative immunoblots of melanogenesis components in SK-MEL-30 cells three and four days post-infection with indicated shRNA lentiviruses (*Scr*, scrambled shRNA control; *A6_1*, shRNA targeting SLC16A6 #1; *A6_2*, shRNA targeting SLC16A6 #2; n=3, biological replicates). *Right*, quantification of relative protein expression of shRNA treatment in at least n=3 biological replicates. Each protein is normalized to control alpha-tubulin (*$P < 0.05$; **$P < 0.01$).
